# Breaking Barriers: Delivering Therapeutics to the Brain in Alzheimer’s Disease

**DOI:** 10.1016/j.tjpad.2025.100229

**Published:** 2025-06-13

**Authors:** Bart De Strooper, Philip Scheltens, Stephen Salloway

**Affiliations:** a Center for Brain and Disease research at VIB@KULeuven, University of Leuven, Belgium and Laboratory of the cellular phase of Alzheimer’s Disease at UKDRI@University College London, UCL, UK.; b EQT Life Sciences, Amsterdam, The Netherlands; c Departments of Neurology and Psychiatry, Warren Alpert Medical School of Brown University, Providence, Rhode Island, USA

Despite major advances in understanding the molecular pathology of neurological disorders, therapeutic progress has lagged behind—particularly for diseases like Alzheimer’s. Antibodies, proteins, and genetic medicines hold tremendous promise, yet their clinical efficacy in brain disorders has been modest at best. A central obstacle is the blood–brain barrier (BBB), a highly selective endothelial interface that prevents over 98 % of small molecules, (> 500 Da), and virtually all large biologics from reaching the brain parenchyma [1].

This barrier, formed by tightly sealed brain capillary endothelial cells and supported by pericytes and astrocytic end-feet, spans an estimated 12–18 m² in the human brain. Its protective function is essential for neural homeostasis—but it comes at a cost. Most Alzheimer’s disease (AD) therapeutics, including monoclonal antibodies like aducanumab, lecanemab, and donanemab, cross the BBB inefficiently. While some reports suggest up to 1 % of administered antibodies reach the brain, rigorous pharmacokinetic analyses more commonly estimate this at 0.01–0.1 % [1,2]. This minimal uptake necessitates high systemic doses, increasing the risk of off-target effects and side effects such as amyloid-related imaging abnormalities (ARIA).

In this issue, Lotte de Koning [7] et al. provide a timely and comprehensive review of emerging strategies to overcome the BBB. Among the three approaches discussed, receptor-mediated transcytosis (RMT) is arguably the most mechanistically grounded. RMT exploits native transport systems—such as the transferrin receptor (TfR1)—to ferry therapeutics across the endothelium. Advances in antibody engineering have made this a tractable strategy, as demonstrated by Roche’s Brainshuttle™ platform. This approach attaches a TfR1-targeting moiety to a therapeutic antibody, enabling efficient uptake and transcytosis without interfering with physiological iron transport.

Other methods reviewed by the authors are more empirical. Focused ultrasound (FUS), which transiently opens the BBB in specific regions using circulating microbubbles and acoustic pressure, was first conceptualized [3] in oncology but is now under investigation in AD. The third strategy, nanocarrier-based delivery systems—including liposomes, nanoparticles, and exosomes—has a longer history, dating back to Juliano and Stamp (1975) [4]. While conceptually appealing, nanocarrier delivery faces persistent hurdles: variability in composition, uncertain release kinetics, and limited targeting precision have so far hindered clinical translation.

These delivery systems must also overcome a second, often underappreciated challenge: cellular uptake and intracellular trafficking. Crossing the BBB is only the first step. Once in the brain, therapeutics must be taken up by the intended cell type and escape endosomal compartments to reach their intracellular targets. For some targets, such as BACE1 [2,5], which resides in endosomes, this may be sufficient. For others—such as antisense oligonucleotides (ASOs) that require cytosolic access—endosomal escape remains a critical bottleneck.

Alternative approaches such as intrathecal or intranasal delivery bypass the BBB altogether but carry their own limitations, including invasiveness, dosing constraints, and limited distribution in the human brain’s larger volume compared to rodents.

Among these strategies, trontinemab—a re-engineered version of gantenerumab—exemplifies the promise of BBB shuttles. By incorporating TfR1-binding modules into a bispecific antibody format, Roche’s Brainshuttle™ enabled dramatic enhancement of brain uptake and rapid amyloid clearance within [6,7] months—at much lower doses than traditional anti-amyloid therapies and with minimal ARIA risk. Trontinemab’s clinical performance contrasts starkly with gantenerumab’s earlier failure, underscoring the transformative potential of improved delivery rather than just better targets.

The TfR1 program with trontinemab is associated with side-effects in administration and chronic use. Infusion reactions are common, affecting up to 50 % of patients and currently require prophylactic treatment with corticosteroids prior to dosing. Trontinemab is also associated with anemia caused by reductions in ferritin which can be manged by oral iron supplementation. It is unclear to what degree these side-effects can be overcome and if not, how might this impact the uptake of this promising treatment in clinical practice?

There are also questions about the pattern of distribution of trontinemab in the brain that may help explain the much lower rates of ARIA. A model has been developed showing diffuse capillary uptake of drug that bypasses early binding to amyloid deposits in meningeal vessels. This hypothesis requires further exploration. We agree with de Koning and colleagues that better quantitative methods are needed to measure the amount and topographic pattern of drug reaching the brain.

The success of the trontinemab TfR1 program has galvanized broader interest. Denali Therapeutics and other groups are developing bispecific antibodies and conjugates for neurodegenerative and lysosomal storage diseases. Some gene and RNA therapies are now being engineered with BBB-targeting moieties to enable systemic delivery, such as the recent attempt to deliver an ASO over the BBB using the TfR receptor (6). These advances hold great promise for future drug development, but more work is needed to develop more brain-specific shuttles, overcoming the peripheral side effects, exploring short and long term safety and determining the right payload for the right indication. Pharma interest seems huge, exemplified by the recent acquisition of Aliada Tx, who developed a p-glu mAb against AD coupled with their own proprietary shuttle, by AbbVie for 1.4B USD (https://news.abbvie.com/2024-12-11-AbbVie-Completes-Acquisition-of-Aliada-Therapeutics?utm_source=chatgpt.com). On the other hand the field is very young. Of the 20+ companies developing shuttled programs against neurological disorders, as of March 2025, only one got FDA approval (IZCARGO, by JCR Pharmaceuticals who developed a shuttled enzyme replacement drug for Hunter Syndrome). All these data however, do suggest that coupling CNS-active agents with BBB transit mechanisms could become a generalizable solution across brain diseases.

As trontinemab enters Phase III trials, the field awaits the key question: will enhanced brain delivery translate into meaningful and durable clinical benefit? If so, it may signal a transformative paradigm shift—not only for AD, but for how we design, deliver, and evaluate therapies in all CNS disorders. What was once an impermeable barrier may soon become a gateway to precision brain therapeutics.

## Declaration of generative AI and AI-assisted technologies in the writing process

During the preparation of this work, Bart de Strooper used Chat GPT 4.0 to improve writing. After using this tool, the authors reviewed and edited the content, as needed, and take full responsibility for the content of the publication.

## CRediT authorship contribution statement

**Bart De Strooper:** Conceptualization, Writing – original draft, Writing – review & editing. **Philip Scheltens:** Conceptualization, Writing – original draft, Writing – review & editing. **Stephen Salloway:** Conceptualization, Writing – original draft, Writing – review & editing.

## Disclosures

BDS has performed consultancies for several large and small companies (including Roche, Eisai, EQTpartners, Remynd, Tactile, Earlybird, Sironax, Montis, Abyssinia) over the past 3 years and is a minor shareholder of Muna Tx.

PhS is a full-time employee of EQT Life Sciences. He is also co-chair of the EVOKE studies of NOVO Nordisk and member of the DSMB of the Retain study and Immunobrain study.

SS provides consultation to Lilly, Biogen, Roche, Genentech, Eisai, Acumen, NovoNordisk, Prothena, Labcorp, AbbVie and Neurophet. He is also an Associate Editor of the Journal of the Prevention of Alzheimer’s Disease. Butler Hospital receives support for clinical trials from Lilly, Biogen, Genentech, Johnson and Johnson, Roche, Eisai and Novartis.
